# Supplementary material for: Transcriptomics and chromatin accessibility in multiple African population samples
Source: bioRxiv. 2023 Nov 6:2023.11.04.564839. Preprint. [Version 1] doi: 10.1101/2023.11.04.564839 (PMC10659267; doi:10.1101/2023.11.04.564839)
Supplement: Supplement 2 [file media-2.zip › biorxiv-AFGR-SupplementaryTables/biorxiv-AFGR-SupplementaryTableS2.pdf]

Supplementary Table 2. Number of transcribed features detected across all AFGR samples. Unnanotated features do not have an annotated in GENCODEv27, and novel features are a subset of the unannotated that are also not detected in long-read RNA-seq of GM12787 or GENCODEv43. African pan-genomic contig features are those for which reads preferentially aligned to the a contig from the pan-genomic contig collection published by Sherman et al.

**Feature**	**Reference genome expression**			**African pan-genomic contig expression**
	Total	Unannotated	Novel	Novel
Exons	310504	57916	43994	2072
Transcripts	108971	26521	25277	367
Loci	21021	1303	248	284
