# Supplementary material for: Transcriptomics and chromatin accessibility in multiple African population samples
Source: bioRxiv. 2023 Nov 6:2023.11.04.564839. Preprint. [Version 1] doi: 10.1101/2023.11.04.564839 (PMC10659267; doi:10.1101/2023.11.04.564839)
Supplement: Supplement 2 [file media-2.zip › biorxiv-AFGR-SupplementaryTables/biorxiv-AFGR-SupplementaryTableS10.pdf]

Supplementary Table 10. Splice Cluster Filtering

Step	Clusters	Introns
Leaf Cutter output (clustered across all African populations)	37,490 (min 2, median 3, max 72 introns/cluster)	151,808
Filter out clusters with more than 10 introns	35,798	124,844
By population, introns supported by at least 10% of total number reads assigned to the cluster in at least 25% of samples, then intersect to set that passes in every population	32,609	56,920
Filter out clusters with fewer than 2 active introns (14,812 single intron clusters)	17,797	42,108
Filter out clusters with low splicing variability, ie, Hellinger's distance < 0.01	13,359	32,493
